# Association between Interleukin-6 Promoter Polymorphism (-174 G/C), Serum Interleukin-6 Levels and Mortality in Severe Septic Patients

**DOI:** 10.3390/ijms17111861

**Published:** 2016-11-08

**Authors:** Leonardo Lorente, María M. Martín, Antonia Pérez-Cejas, Ysamar Barrios, Jordi Solé-Violán, José Ferreres, Lorenzo Labarta, César Díaz, Alejandro Jiménez

**Affiliations:** 1 Intensive Care Unit, Hospital Universitario de Canarias, Ofra, s/n, La Laguna, 38320 Santa Cruz de Tenerife, Spain; 2 Intensive Care Unit, Hospital Universitario Nuestra Señora Candelaria, Crta Rosario s/n, 38010 Santa Cruz de Tenerife, Spain; mar.martinvelasco@gmail.com; 3 Laboratory Deparment, Hospital Universitario de Canarias, Ofra, s/n, La Laguna, 38320 Santa Cruz de Tenerife, Spain; aperezcejas@gmail.com; 4 Research Unit, Hospital Universitario de Canarias, Ofra, s/n, La Laguna, 38320 Santa Cruz de Tenerife, Spain; ysamar.barrios@gmail.com; 5 Intensive Care Unit, Hospital Universitario Dr. Negrín, Barranco de la Ballena s/n, 35010 Las Palmas de Gran Canaria, Spain; jsolvio@gobiernodecanarias.org; 6 Intensive Care Unit, Hospital Clínico Universitario de Valencia, Avda, Blasco Ibáñez nº17, 46004 Valencia, Spain; ferreresj@gmail.com; 7 Intensive Care Unit, Hospital San Jorge de Huesca, Avenida Martínez de Velasco nº36, 22004 Huesca, Spain; llabarta@salud.aragon.es; 8 Intensive Care Unit, Hospital Insular, Plaza Dr. Pasteur s/n, 35016 Las Palmas de Gran Canaria, Spain; incaicos@yahoo.es; 9 Research Unit, Hospital Universitario de Canarias, Ofra, s/n, La Laguna-38320, 38320 Santa Cruz de Tenerife, Spain; ajimenezsosa@gmail.com

**Keywords:** IL-6, 174 G/C, sepsis, mortality, outcome, prognosis

## Abstract

The association between interleukin (IL)-6 promoter polymorphism (-174 G/C), circulating IL-6 levels and mortality in septic patients has scarcely been addressed, and then only in studies of small sample size, and a direct association among them has not been previously reported. Therefore, the purpose of our study was to determine whether this association exists. An observational, prospective and multicenter study including severe septic patients was undertaken and serum IL-6 levels at severe sepsis diagnosis and IL-6 promoter polymorphism (-174 G/C) were determined. The end-point of the study was 30-day mortality. The study included 263 patients with the following genotypes of IL-6 promoter polymorphism (-174 G/C): 123 (46.8%) GG, 110 (41.8%) GC and 30 (11.4%) CC. CC homozygous patients showed lower sepsis-related organ failure assessment (SOFA) score, serum IL-6 levels and mortality at 30 days compared to those with other genotypes (GC or GG). On regression analysis, CC homozygous patients showed lower 30-day mortality than those with genotype GG (odds ratio = 0.21; 95% CI = 0.053−0.838; *p* = 0.03) or GC (hazard ratio = 0.28; 95% CI = 0.074−1.037; *p* = 0.06). The most important results of our study were that CC might be a favorable genotype in septic patients showing lower serum IL-6 levels and lower risk of death within 30 days.

## 1. Introduction

Sepsis is a major cause of mortality and resource consumption [1,2]. Interleukin (IL)-6 is one of several pro-inflammatory cytokines involved in systemic response [3]. Higher circulating IL-6 levels have been reported in non-surviving rather than surviving septic patients [4,5,6,7,8].

An association between IL-6 promoter polymorphism (-174 G/C) and prognosis has been found in different diseases [9,10,11,12,13]. In addition, an association between IL-6 promoter polymorphism (-174 G/C) and circulating IL-6 levels has been found in different diseases [11,12,13,14,15,16,17]. The influence of IL-6 promoter polymorphism (-174 G/C) on sepsis risk and sepsis-related mortality has been addressed in some studies [18,19,20,21,22,23,24,25,26,27,28,29], but a recently published meta-analysis found no association between this polymorphism and sepsis-related mortality [30]. However, in most of those studies the sample size was small and circulating levels of IL-6 were not reported. Therefore, the purpose of our study was to determine whether there is an association between IL-6 promoter polymorphism (-174 G/C), serum levels of IL-6 and 30-day mortality in a large series of adult septic patients.

## 2. Results

The study included 263 severe septic patients with the following genotypes of IL-6 promoter polymorphism (-174 G/C): 123 (46.8%), with genotype GG, 110 (41.8%) with genotype GC and 30 (11.4%) with genotype CC. No significant difference in Hardy-Weinberg equilibrium was found between the genotypes of our series (chi-square = 0.20; *p* = 0.66).

**Figure 1:**
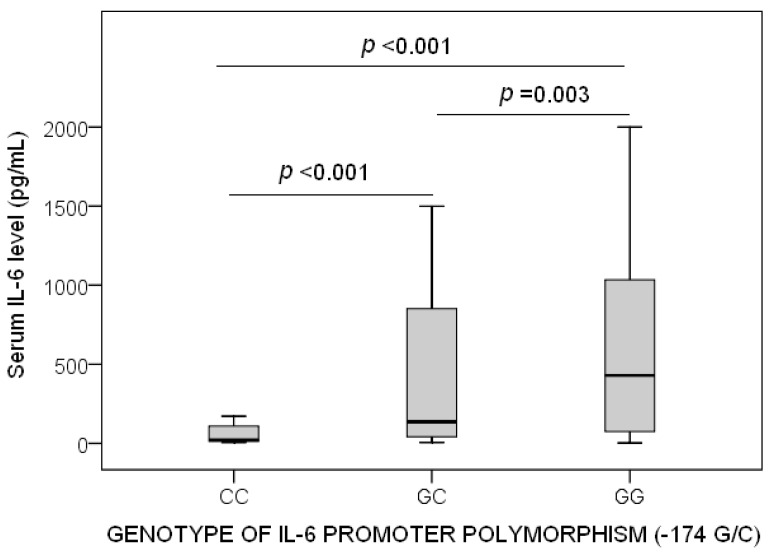
Serum interleukin-6 levels according to the genotype of IL-6 promoter polymorphism (-174 G/C).

We found that 223 (84.8%) patients showed septic shock, that in 132 (50.2%) patients all cultures were sterile, and that 86 (32.7%) patients were dead at 30 days. We do not find statistically significant differences between patient groups in sex, age, diabetes mellitus, ischemic heart disease, chronic obstructive pulmonary disease (COPD), microorganism responsible, site of infection, bloodstream infection, empiric antimicrobial treatment, septic shock at the time of enrolment in the study, creatinine, leukocyte count, bilirubin, lactic acid, platelets, international normalized ratio (INR), and activated partial thromboplastin time (aPTT). However, CC homozygous patients showed higher PaO_2_/FIO_2_, and lower sepsis-related organ failure assessment (SOFA) score, serum IL-6 levels and mortality at 30 days (Table 1). All cases of 30-day mortality were due to sepsis. In addition, we found that CC homozygous patients showed lower serum IL-6 levels than patients with genotypes GG (*p* < 0.001) or GC (*p* < 0.001), and that patients with genotypes GC showed lower serum IL-6 levels than patients with genotypes GG (*p* = 0.003) (Figure 1).

**Figure 2:**
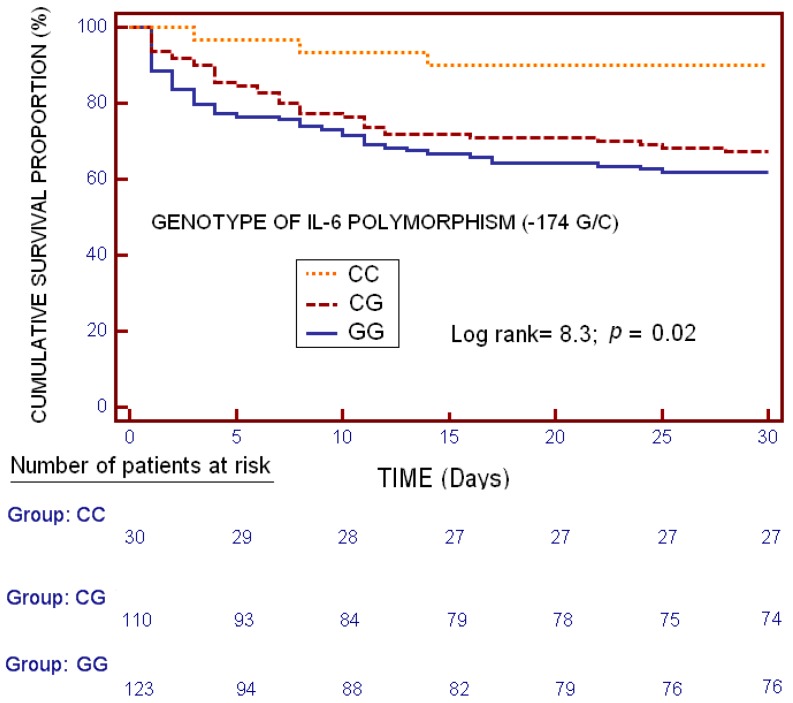
Kaplan-Meier curves showing the cumulative proportion of surviving patients at 30 days according to the presence of CC vs. other genotypes (GG or GC) of IL-6 promoter polymorphism (-174 G/C).

Kaplan-Meier analysis showed differences in mortality at 30 days between different genotypes (*p* = 0.02) (Figure 2). We found that CC homozygous patients showed lower risk of mortality at 30 days than patients with genotypes GC (hazard ratio = 0.18; 95% CI = 0.05−0.62) or GG (hazard ratio = 0.23; 95% CI = 0.06−0.80), and we do not find statistically significant differences between GC and GG genotypes (hazard ratio = 0.78; 95% CI = 0.46−1.35).

On regression analysis, CC homozygous patients showed lower 30-day mortality than those with genotype GG (odds ratio = 0.21; 95% CI = 0.053−0.838; *p* = 0.03) or GC (hazard ratio = 0.28; 95% CI = 0.074−1.037; *p* = 0.06) after controlling for age, baseline measures of SOFA score and serum lactic acid levels (Table 2). In addition, we found that serum IL-6 levels were associated with higher mortality at 30 days (odds ratio = 1.001; 95% CI = 1.0001−1.001; *p* = 0.002) after controlling for age, baseline measures of SOFA score and serum lactic acid levels.

## 3. Discussion

The most important results of our study were that septic patients with genotype CC in IL-6 promoter polymorphism (-174 G/C) had lower serum IL-6 levels and lower risk of death within 30 days than those with GC or GG genotypes. To our knowledge, this is the largest series reporting data about IL-6 promoter polymorphism (-174 G/C), serum IL-6 levels and early mortality in septic patients.

The influence of IL-6 promoter polymorphism (-174 G/C) on sepsis risk and sepsis mortality has been addressed in some studies [18,19,20,21,22,23,24,25,26,27,28,29]. A recently published meta-analysis found no association between IL-6 promoter polymorphism (-174 G/C) and sepsis-related mortality [30]. In addition, another two studies were published after the meta-analysis [18,19]. In children with community-acquired pneumonia (CAP), Zidan et al. found that patients with genotype GG showed a lower risk of early mortality [19]. In 277 Chinese patients with severe pneumonia-induced sepsis, Feng et al. found no association between IL-6 promoter polymorphism (-174 G/C) and mortality [18]. Most previous studies had a small sample size (fewer than 200 patients) and circulating levels of IL-6 were not reported. Only three studies had a sample size higher than 200 patients (277, 306 and 1135 patients, respectively) [18,22,23]. Rodriguez-Gallego et al. did not find an association between that polymorphism and mortality in patients with CAP [23]; and in another study, the same team found that pneumococcal CAP patients with genotype GG had a lower risk of death [22]. Our finding of lower mortality in CC homozygous severe septic patients contradict those of Rodriguez-Gallego et al. [22] and Feng et al. [18]. The discrepancy between study findings could be due to differences in ethnicity (European, African, Asian), age (adult, pediatric, neonatal), type of infection (sepsis, CAP, meningococcemia, pneumococcal infection), and severity of infection (sepsis, severe sepsis, septic shock). Rodriguez-Gallego et al. found that patients with genotype GG had a lower risk of early death [22]; their study included patients with pneumococcal CAP who had a rate of septic shock of 19.9%, and 5% mortality at 28 days. Feng et al. did not find an association between IL-6 promoter polymorphism (-174 G/C) and mortality [18]; they included patients with severe pneumonia-induced sepsis, mean SOFA score of 5 points, microorganism responsible for pneumonia isolated in 38.6%, a rate of septic shock of 34.7%, and a mortality rate of 22.7%. Our study included patients with severe sepsis with different sites of infection (55% respiratory, 29% abdominal and 16% others), mean SOFA score of 9 points, microorganism responsible for sepsis isolated in 50.2%, rate of septic shock of 84.7%, and 33% of patients had died at 30 days. Our finding of lower early mortality in CC homozygous severe septic patients are in consonance with that of Balding et al. who studied 183 children with meningococcemia and they too found lower early mortality in CC homozygous patients [29]. In addition, other authors have reported better prognosis in CC homozygous patients of IL-6 promoter polymorphism (-174 G/C) in various diseases, such us different types of cancer, surgical coronary revascularization and end-stage renal disease on hemodialysis [10,11,12,13,14].

An interesting finding in our series of severe septic patients was the association between IL-6 promoter polymorphism (-174 G/C) and serum levels of IL-6. This association has scarcely been addressed in septic patients [19,21,26,31], and then only in studies of small sample size, the largest being 112 severe septic patients in a study by Tischendorff et al. [31]. Two of the above studies reported higher circulating levels of IL-6 in patients with genotype GG [26,31]; however, an association between IL-6 promoter polymorphism (-174 G/C) and survival was not found. In addition, another two of those studies found higher early mortality in GG patients than in those with other genotypes [19,21]; however, they did not find an association between IL-6 promoter polymorphism (-174 G/C) and circulating levels of IL-6. Thus, our finding of lower serum IL-6 levels in patients with CC genotype than in those with other genotypes is in consonance with that previously reported [26,31], and our study included a higher sample size (263 patients). In addition, other studies have reported lower circulating levels of IL-6 in CC homozygous patients with different types of cancer, arthritis, cirrhosis, pancreatitis, retinal idiopathic Eales’s vasculitis, surgical coronary revascularization and end-stage renal disease on hemodialysis [12,13,14,15,16,17,18].

Another interesting finding of our study was the association between circulating IL-6 levels and early mortality in severe septic patients. We found higher serum IL-6 levels in non-survivors than in surviving patients, in agreement with previous studies [5,6,7,8,9].

To our knowledge, this is the first study to report an association between IL-6 promoter polymorphism (-174 G/C), circulating levels of IL-6 and early mortality in septic patients. As previously mentioned, other authors have reported better prognosis in CC homozygous patients, including children with meningococcemia [29] and adults with non-infectious diseases [10,11,12,13,14]. Also, lower circulating levels of IL-6 have been found in CC homozygous patients with sepsis [26,31] and other non-infectious diseases [12,13,14,15,16,17,18]. Lastly, lower serum IL-6 levels have been reported in non-surviving than surviving septic patients [5,6,7,8,9]. We found that the lower 30-day mortality rate and serum IL-6 levels were presented by patients with genotype CC, followed by GC patients and finally by GG patients. Thus, a possible explanation for our findings is that CC homozygous patients had lower circulating levels of IL-6; they therefore presented a lower inflammatory response with lower sepsis severity (reflected by a lower SOFA score) and finally, lower risk of death. There are contradictory reports about the role of IL-6 polymorphisms in infectious diseases. The inflammatory response, particularly IL-6 production, varies greatly depending on the causative pathogen and the route of infection [32,33]. In the context of compartmentalized infections, as in many patients with pneumococcal pneumonia, the IL-6 -174 GG genotype (associated with higher IL-6 production) may be protective against severe complications, as previously reported by Rodriguez-Gallego et al. [22]. In contrast, the CC genotype (associated with lower IL-6 production) may be protective against inflammatory response in severe septic patients, according to the results of our study.

The frequencies of the IL-6 promoter polymorphism (-174 G/C) genotypes in our septic patients (46.8% with GG, 41.8% with GC and 11.4% with CC genotype) are similar to those found by Rodriguez-Gallego et al. (46.8% with GG, 42.6% with GC and 10.5% with CC genotype) [23].

Our study has certain limitations. First, we determined only one genetic polymorphism. It is difficult that a single nucleotide polymorphism (SNP) of a proinflammatory cytokine gene might be relevant for critical patient outcome [34]; however, our sample size was sufficiently large to show an association between the IL-6 promoter polymorphism (-174 G/C) and early mortality. Regardless, the determination of genetic polymorphisms of other cytokines (such as tumor necrosis factor, IL-1, IL-10) could be interesting; Second, we did not determine IL-6 promoter polymorphism (-174 G/C) in non-septic critically ill patients or healthy control subjects; however, our purpose was to determine whether there is an association between the polymorphism and sepsis survival, not to analyze the association between the polymorphism and the appearance of sepsis; Third, the sample size in the group of patients with the CC genotype was low; however, it was sufficiently large to show an association between the IL-6 promoter polymorphism (-174 G/C), serum IL-6 levels and early mortality; Fourth, we did not report data on treatments and treatment response over time; Fifth, our study cohort was a heterogeneous group of septic patients with different sites of infection and microorganisms responsible for infection; Sixth, we did not record the exact moment when blood samples were obtained, nor the time interval between the onset of symptoms and blood sampling; Seventh, blood samples were immediately stored at −80 °C until the determinations; however, they were not placed on ice during the time to storage; Eighth, we have not collected the number of patients removed from the study due to different causes (patients declining participation, more than two hours since the diagnosis, exclusion criteria, etc.).

## 4. Materials and Methods

### 4.1. Design and Subjects

A multicenter, prospective and observation study was carried out between 2008–2009 with 263 severe septic patients from six Intensive Care Units of Spain. Institutional Review Boards of the following six participating hospitals approved the study: Hospital Insular (Las Palmas de Gran Canaria), Hospital Clínico Universitario de Valencia (Valencia), Hospital Universitario de Canarias (La Laguna. Tenerife), Hospital Universitario Dr. Negrín (Las Palmas de Gran Canaria), Hospital Universitario Nuestra Señora de Candelaria (Santa Cruz de Tenerife), Hospital San Jorge (Huesca). Written informed consent was obtained from patients or their family members.

We included patients diagnosed with septic shock or severe sepsis according to the International Sepsis Definitions Conference [35]. We excluded patients with steroid, immunosuppressive or radiation therapy, hematologic or solid tumor, white blood cell count <1000/mm^3^, human immunodeficiency virus (HIV), age <18 years, pregnancy, or lactation.

The same cohort of severe septic patients was used in previous publications by our team for other objectives [36,37,38,39]. In the current research, we sought to determine whether there is an association between IL-6 promoter polymorphism (-174 G/C), serum levels of IL-6 and 30-day mortality in a large series of adult septic patients.

### 4.2. Variables Recorded

Bloodstream infection, diabetes mellitus, chronic obstructive pulmonary disease (COPD), empiric antimicrobial treatment, ischemic heart disease, microorganism responsible, sex, site of infection, age, bilirubin, activated partial thromboplastin time (aPTT), sepsis-related organ failure assessment (SOFA])score [40], international normalized ratio (INR), lactic acid, creatinine, leukocytes, platelets, fraction inspired of oxygen (FiO_2_), pressure of arterial oxygen (PaO_2_) were recorded for all patients. The end-point of the study was 30-day mortality. We used serum separator tubes (SST) for the determination of bilirubin and creatinine, citrated plasma tubes for the determination of activated partial thromboplastin time (aPTT) and international normalized ratio (INR), and ethylenediaminetetraacetic acid (EDTA)-containing tubes for the determination of leukocyte and platelet counts.

Empiric antimicrobial therapy was considered adequate if the microorganism responsible for sepsis was susceptible to at least one of the antimicrobial agents used, inadequate if the microorganism responsible for sepsis was not susceptible to any antimicrobial agent used, and unknown whether adequate when it was not possible to know whether the microorganism responsible for sepsis was susceptible to any antimicrobial agent used (in cases of sterile cultures or diagnosis by antigenuria).

### 4.3. Blood Samples and Determinations

We collected venous blood samples at diagnosis of severe sepsis (within the first two hours of diagnosis of severe sepsis) for the determination of serum IL-6 concentration and genetic polymorphism of IL-6, and the samples were immediately stored at −80 °C until the determinations.

The determination of IL-6 promoter polymorphism (-174 G/C) (rs1800795) was carried out in the Research Unit of Hospital Universitario de Canarias (La Laguna, Santa Cruz de Tenerife, Spain). We performed genotyping by polymerase chain reactions (PCR) and restriction fragment length polymorphism (RFLP) analysis. We prepared DNA from 3 mL of peripheral blood using treatment with proteinase K, extraction of phenol-chloroform extraction and precipitation of ethanol. We used approximately 100 ng DNA as the template in PCR using the following primers, flanking the -174 G/C polymorphism (rs1800795) of the *IL-6* gene: 5′-TTGTCAAGACATGCCAAAGTG-3′ and 5′-TCAGACATCTCCAGTCCTATA-3′, and the temperature profile: 94C-52C-72C, 30 s each, for 30 cycles. We restricted the amplified DNA with endonuclease *NIa III (CATG)* (New England Biolabs, Boston, MA, USA) for two hours at 37 °C. The resulting DNA fragments were separated by gel electrophoresis in 2% agarose gel and visualized under ultraviolet light. In the absence of a *NlaIII* site, a fragment of 300 bp was detected (G allele), whereas fragments of 169 and 131 bp corresponded to the C allele.

The determination of serum levels of IL-6 concentrations was carried out in the Laboratory Department of Hospital Universitario de Canarias (La Laguna, Santa Cruz de Tenerife, Spain) using the kit IMMULITE (Siemens Healthcare Diagnostics Products Ltd., LLanberis, Gwynedd, UK), which is a solid-phase, enzyme-labeled, chemiluminescent sequential immunometric assay. The inter-assay coefficient of variation (CV) was 5.1%–7.5%, the intra-assay CV was 3.5%–6.2%; and the limit of detection was 2 pg/mL.

### 4.4. Statistical Methods

We recorded categorical variables as frequencies and percentages, and we compared them between groups by chi-square test. We recorded continuous variables as medians and interquartile ranges, and we compared them between groups by Kruskall-Wallis test. To determine the association of IL-6 promoter polymorphism (-174 G/C) with mortality at 30 days (controlling for SOFA score, serum lactic acid levels and age) a confirmatory multivariate logistic regression analysis with comparisons of pair to pair genotypes was used. In addition, we also carried out a multivariate logistic regression analysis to determine the association of serum IL-6 levels with mortality at 30 days after controlling for SOFA score, serum lactic acid levels and age. The clinical impact for the predictor variables was calculated by odds ratio (OR) and 95% confidence intervals (CI). We plotted 30-day survival curves of patient groups with CC, GC and GG genotypes using the Kaplan-Meier method and we compared them using log-rank test. We tested the Hardy-Weinberg equilibrium in the genotypes of our series using the chi-square test. P-values of less than 0.05 were considered statistically significant. NCSS 2000 (Kaysville, UT, USA) and SPSS 17.0 (SPSS Inc., Chicago, IL, USA) were used for statistical analyses.

## 5. Conclusions

To our knowledge, the present study involved the largest sample size to date and is the first to report an association between IL-6 promoter polymorphism (-174 G/C), serum IL-6 levels and early mortality in septic patients. The most important results of our study were that CC might be a favorable genotype in septic patients showing lower serum IL-6 levels and lower risk of death within 30 days.

## Figures and Tables

**Table 1:** Characteristics of septic patients according to genotype of IL-6 promoter polymorphism (-174 G/C).

Demographic and Clinical Characteristics	GG (*n* = 123)	GC (*n* = 110)	CC (*n* = 30)	*p*-Value
Bloodstream infection—*n* (%)	17 (13.8)	22 (20.0)	3 (10.0)	0.28
COPD—*n* (%)	15 (12.2)	14 (12.7)	6 (20.0)	0.52
Diabetes mellitus—*n* (%)	39 (31.7)	34 (30.9)	6 (20.0)	0.44
Empiric antimicrobial treatment—*n* (%)	–	–	–	0.28
Unknown whether adequate due to negative cultures	63 (51.2)	52 (47.3)	17 (56.7)	–
Unknown whether adequate due to diagnosis by antigenuria	5 (4.1)	1 (0.9)	1 (3.3)	–
Adequate	45 (36.6)	52 (47.3)	11 (36.7)	–
Inadequate	11 (8.9)	4 (3.6)	1 (3.3)	–
Female gender—*n* (%)	43 (35.0)	35 (31.8)	12 (40.0)	0.69
Ischemic heart disease—*n* (%)	16 (13.0)	9 (8.2)	4 (13.3)	0.46
Mechanical ventilation—*n* (%)	104 (84.6)	95 (86.3)	24 (80.0)	0.51
Microorganism responsible—*n* (%)	–	–	–	–
Unknown	63 (51.2)	52 (47.3)	17 (56.7)	0.63
Gram-positive	28 (22.8)	30 (27.3)	7 (23.3)	0.72
Gram-negative	32 (26.0)	27 (24.5)	8 (26.7)	0.96
Fungii	6 (4.9)	1 (0.9)	0	0.11
Anaerobe	1 (0.8)	1 (0.9)	0	0.88
Mortality at 30 days—*n* (%)	47 (38.2)	36 (32.7)	3 (10.0)	0.01
Septic shock—*n* (%)	104 (84.6)	95 (87.2)	24 (80.0)	0.60
Site of infection—*n* (%)	–	–	–	0.84
Respiratory	64 (52.0)	67 (60.9)	14 (46.7)	–
Abdominal	39 (31.7)	28 (25.5)	10 (33.3)	–
Neurological	4 (3.3)	1 (0.9)	0	–
Urinary	6 (4.9)	5 (4.5)	2 (6.7)	–
Skin	5 (4.1)	4 (3.6)	2 (6.7)	–
Endocarditis	5 (4.1)	4 (3.6)	2 (6.7)	–
Osteomyelitis	0	1 (0.9)	0	–
Age—median years (p_25–75_)	56 (43–68)	61 (50–70)	63 (51–78)	0.32
APACHE-II score—median (p_25–75_)	20 (15–25)	21 (16–25)	20 (17–22)	0.62
aPTT (seconds)—median (p_25–75_)	35 (29–44)	33 (28–43)	28 (27–43)	0.09
Bilirubin (mg/dl)—median (p_25–75_)	0.90 (0.50–2.49)	0.80 (0.41–1.50)	0.80 (0.51–1.63)	0.20
Creatinine (mg/dl)—median (p_25–75_)	1.30 (0.70–2.28)	1.40 (0.90–2.45)	1.60 (0.90–2.55)	0.94
Hospitalization before enrollment in the study (days)—median (p_25–75_)	0 (0–5)	0 (0–5)	0 (0–3)	0.20
GCS—median (p_25–75_)	15 (13–15)	15 (8–15)	14 (10–15)	0.40
Interleukin-6 levels (pg/mL)—median (p_25-75_)	429 (74–1034)	136 (41–852)	21 (13–116)	<0.001
INR—median (p_25–75_)	1.42 (1.20–1.71)	1.20 (1.06–1.50)	1.36 (1.11–1.50)	0.0033
Lactic acid (mmol/L)—median (p_25–75_)	2.15 (1.13–4.38)	2.20 (1.25–4.45)	2.00 (1.15–3.15)	0.11
Leukocytes (cells/mm^3^)—median × 10^3^ (p_25–75_)	14.1 (8.9–20.1)	15.2 (7.9–22.0)	16.2 (13.4–22.4)	0.57
Pa0_2_/FI0_2_ ratio—median (p_25–75_)	194 (111–265)	160 (102–221)	250 (172–298)	0.001
Platelets (cells/mm^3^)—median × 10^3^ (p_25–75_)	165 (90–243)	179 (101–270)	210 (93–267)	0.17
SOFA score—median (p_25−75_)	10 (7–13)	10 (7–12)	8 (6–11)	0.02

COPD = chronic obstructive pulmonary disease; APACHE = Acute physiology and chronic health evaluation; aPTT = Activated partial thromboplastin time; GCS = Glasgow coma scale; INR = International normalized ratio; PaO_2_/FIO_2_ = pressure of arterial oxygen/fraction inspired oxygen; SOFA = Sepsis-related organ failure assessment; p_25–75_ = percentile_25–75_.

**Table 2:** Multiple logistic regression analyses to predict 30-day mortality.

Predictors	Odds Ratio	95% Confidence Interval	*p*-Value
**First Model**	–	–	–
SOFA (points)	1.15	1.023–1.284	0.02
Lactic acid levels (mmol/L)	1.23	1.063–1.430	0.006
Age (years)	1.02	0.990–1.042	0.23
Genotype of interleukin-6 (CC vs. GG)	0.21	0.053–0.838	0.03
**Second Model**	–	–	–
SOFA (points)	1.22	1.068–1.395	0.003
Lactic acid levels (mmol/L)	1.08	0.933–1.254	0.30
Age (years)	1.02	0.988–1.052	0.22
Genotype of interleukin-6 (CC vs. GC)	0.28	0.074–1.037	0.057
**Third Model**	–	–	–
SOFA (points)	1.16	1.062–1.263	0.001
Lactic acid levels (mmol/L)	1.14	1.029–1.033	0.01
Age (years)	1.01	0.993–1.033	0.22
Serum interleukin-6 levels (pg/mL)	1.001	1.0001–1.001	0.002

## Abbreviations

SOFA	Sepsis-related organ failure assessment
COPD	Chronic obstructive pulmonary disease
aPTT	Activated partial thromboplastin time
IL	Interleukin
INR	International normalized ratio
PaO2	Pressure of arterial oxygen
FIO2	Fraction inspired oxygen

## References

[1] Vincent J.L., Sakr Y., Sprung C.L., Ranieri V.M., Reinhart K., Gerlach H., Moreno R., Carlet J., Le Gall J.R., Payen D., Sepsis occurrence in acutely III patients investigators (2006). Sepsis in European intensive care units: Results of the SOAP study. Crit. Care Med..

[2] Angus D.C., Linde-Zwirble W.T., Lidicker J., Clermont G., Carcillo J., Pinsky M.R. (2001). Epidemiology of severe sepsis in the United States: Analysis of incidence, outcome, and associated costs of care. Crit. Care Med..

[3] Samraj R.S., Zingarelli B., Wong H.R. (2013). Role of biomarkers in sepsis care. Shock.

[4] Hack C.E., de Groot E.R., Felt-Bersma R.J., Nuijens J.H., Strack Van Schijndel R.J., Eerenberg-Belmer A.J., Thijs L.G., Aarden L.A. (1989). Increased plasma levels of interleukin-6 in sepsis. Blood.

[5] Patel R.T., Deen K.I., Youngs D., Warwick J., Keighley M.R. (1994). Interleukin 6 is a prognostic indicator of outcome in severe intra-abdominal sepsis. Br. J. Surg..

[6] Damas P., Ledoux D., Nys M., Vrindts Y., de Groote D., Franchimont P., Lamy M. (1992). Cytokine serum level during severe sepsis in human IL-6 as a marker of severity. Ann. Surg..

[7] Miguel-Bayarri V., Casanoves-Laparra E.B., Pallás-Beneyto L., Sancho-Chinesta S., Martín-Osorio L.F., Tormo-Calandín C., Bautista-Rentero D. (2012). Prognostic value of the biomarkers procalcitonin, interleukin-6 and C-reactive protein in severe sepsis. Med. Intensiv..

[8] Tschaikowsky K., Hedwig-Geissing M., Braun G.G., Radespiel-Troeger M. (2011). Predictive value of procalcitonin, interleukin-6, and C-reactive protein for survival in postoperative patients with severe sepsis. J. Crit. Care.

[9] DeMichele A., Martin A.M., Mick R., Gor P., Wray L., Klein-Cabral M., Athanasiadis G., Colligan T., Stadtmauer E., Weber B. (2003). Interleukin-6-174G>C polymorphism is associated with improved outcome in high-risk breast cancer. Cancer Res..

[10] Hefler L.A., Grimm C., Ackermann S., Malur S., Radjabi-Rahat A.R., Leodolter S., Beckmann M.W., Zeillinger R., Koelbl H., Tempfer C.B. (2003). An interleukin-6 gene promoter polymorphism influences the biological phenotype of ovarian cancer. Cancer Res..

[11] Burzotta F., Iacoviello L., di Castelnuovo A., Glieca F., Luciani N., Zamparelli R., Schiavello R., Donati M.B., Maseri A., Possati G. (2001). Relation of the -174 G/C polymorphism of interleukin-6 to interleukin-6 plasma levels and to length of hospitalization after surgical coronary revascularization. Am. J. Cardiol..

[12] Gomes M., Coelho A., Araújo A., Azevedo A., Teixeira A.L., Catarino R., Medeiros R. (2015). IL-6 polymorphism in non-small cell lung cancer: A prognostic value?. Tumour Biol..

[13] Balakrishnan V.S., Guo D., Rao M., Jaber B.L., Tighiouart H., Freeman R.L., Huang C., King A.J., Pereira B.J., HEMO Study Group (2004). Cytokine gene polymorphisms in hemodialysis patients: Association with comorbidity, functionality, and serum albumin. Kidney Int..

[14] Sen A., Paine S.K., Chowdhury I.H., Mukherjee A., Choudhuri S., Saha A., Mandal L.K., Bhattacharya B. (2011). Impact of interleukin-6 promoter polymorphism and serum interleukin-6 level on the acute inflammation and neovascularization stages of patients with Eales’disease. Mol. Vis..

[15] Giannitrapani L., Soresi M., Giacalone A., Campagna M.E., Marasà M., Cervello M., Marasà S., Montalto G. (2011). IL-6 -174G/C polymorphism and IL-6 serum levels in patients with liver cirrhosis and hepatocellular carcinoma. OMICS.

[16] Talar-Wojnarowska R., Gasiorowska A., Smolarz B., Romanowicz-Makowska H., Kulig A., Malecka-Panas E. (2009). Clinical significance of interleukin-6 (*IL-6*) gene polymorphism and IL-6 serum level in pancreatic adenocarcinoma and chronic pancreatitis. Dig. Dis. Sci..

[17] Fishman D., Faulds G., Jeffery R., Mohamed-Ali V., Yudkin J.S., Humphries S., Woo P. (1998). The effect of novel polymorphisms in the interleukin-6 (*IL-6*) gene on IL-6 transcription and plasma IL-6 levels, and an association with systemic-onset juvenile chronic arthritis. J. Clin. Investig..

[18] Feng B., Mao Z.R., Pang K., Zhang S.L., Li L. (2015). Association of tumor necrosis factor α-308G/A and interleukin-6 -174G/C gene polymorphism with pneumonia-induced sepsis. J. Crit. Care.

[19] Zidan H.E., Elbehedy R.M., Azab S.F. (2014). IL6-174 G/C gene polymorphism and its relation to serum IL6 in Egyptian children with community-acquired pneumonia. Cytokine.

[20] Abdel-Hady H., El-Naggar M., El-Nady G., Badr R., El-Daker M. (2009). Genetic polymorphisms of IL-6-174 and IL-10–1082 in full term neonates with late onset blood stream infections. J. Pediatr. Infect. Dis..

[21] Schluter B., Raufhake C., Erren M., Schotte H., Kipp F., Rust S., Van Aken H., Assmann G., Berendes E. (2002). Effect of interleukin-6 promoter polymorphism (-174 G/C) on the incidence and outcome of sepsis. Crit. Care Med..

[22] Martin-Loeches I., Sole-Violan J., Rodriguez de Castro F., Garcia-Laorden M.I., Borderias L., Blanquer J., Rajas O., Briones M.L., Aspa J., Herrera-Ramos E. (2012). Variants at the promoter of the interleukin-6 gene are associated with severity and outcome of pneumococcal community-acquired pneumonia. Intensive Care Med..

[23] Sole-Violan J., Rodriguez de Castro F., Garcia-Laorden M.I., Blanquer J., Aspa J., Borderías L., Briones M.L., Rajas O., Carrondo I.M., Marcos-Ramos J.A. (2010). Genetic variability in the severity and outcome of community-acquired pneumonia. Respir. Med..

[24] Sipahi T., Pocan H., Akar N. (2006). Effect of various genetic polymorphisms on the incidence and outcome of severe sepsis. Clin. Appl. Thromb. Hemost..

[25] Baier R.J., Loggins J., Yanamandra K. (2006). IL-10, IL-6 and CD14 polymorphisms and sepsis outcome in ventilated very low birth weight infants. BMC Med..

[26] Sabelnikovs O., Nikitina-Zake L., Vanags I. (2008). Association of interleukin 6 promoter polymorphism (-174G/C) with IL-6 level and outcome in severe sepsis. Proc. Latv. Acad. Sci. Sect. B Nat. Exact Appl. Sci..

[27] Shimada T., Oda S., Sadahiro T., Nakamura M., Hirayama Y., Watanabe E., Abe R., Nakada T.A., Tateishi Y., Otani S. (2011). Outcome prediction in sepsis combined use of genetic polymorphisms—A study in Japanese population. Cytokine.

[28] Barber R.C., Aragaki C.C., Rivera-Chavez F.A., Purdue G.F., Hunt J.L., Horton J.W. (2004). TLR4 and TNF-alpha polymorphisms are associated with an increased risk for severe sepsis following burn injury. J. Med. Genet..

[29] Balding J., Healy C.M., Livingstone W.J., White B., Mynett-Johnson L., Cafferkey M., Smith O.P. (2003). Genomic polymorphic profiles in an Irish population with meningococcaemia: Is it possible to predict severity and outcome of disease?. Genes Immun..

[30] Gao J.W., Zhang A.Q., Pan W., Yue C.L., Zeng L., Gu W., Jiang J. (2015). Association between IL-6-174G/C polymorphism and the risk of sepsis and mortality: A systematic review and meta-analysis. PLoS ONE.

[31] Tischendorf J.J., Yagmur E., Scholten D., Vidacek D., Koch A., Winograd R., Gressner A.M., Trautwein C., Wasmuth H.E., Lammert F. (2007). The interleukin-6 (IL6)-174 G/C promoter genotype is associated with the presence of septic shock and the ex vivo secretion of IL6. Int. J. Immunogenet..

[32] Feezor R.J., Oberholzer C., Baker H.V., Novick D., Rubinstein M., Moldawer L.L., Pribble J., Souza S., Dinarello C.A., Ertel W. (2003). Molecular characterization of the acute inflammatory response to infections with gram-negative versus gram-positive bacteria. Infect. Immun..

[33] Yu S.L., Chen H.W., Yang P.C., Peck K., Tsai M.H., Chen J.J., Lin F.Y. (2004). Differential gene expression in gram-negative and gram-positive sepsis. Am. J. Respir. Crit. Care Med..

[34] Accardo Palumbo A., Forte G.I., Pileri D., Vaccarino L., Conte F., D'Amelio L., Palmeri M., Triolo A., D’Arpa N., Scola L. (2012). Analysis of IL-6, IL-10 and IL-17 genetic polymorphisms as risk factors for sepsis development in burned patients. Burns.

[35] Dellinger R.P., Levy M.M., Rhodes A., Annane D., Gerlach H., Opal S.M., Sevransky J.E., Sprung C.L., Douglas I.S., Jaeschke R. (2013). Surviving Sepsis Campaign Guidelines Committee including the Pediatric Subgroup. Surviving sepsis campaign: International guidelines for management of severe sepsis and septic shock: 2012. Crit. Care Med..

[36] Lorente L., Martín M.M., González-Rivero A.F., Ferreres J., Solé-Violán J., Labarta L., Díaz C., Jiménez A., Borreguero-León J.M. (2014). Serum levels of caspase-cleaved cytokeratin-18 and mortality are associated in severe septic patients: Pilot study. PLoS ONE.

[37] Lorente L., Martín M.M., Almeida T., Abreu-González P., Ferreres J., Solé-Violán J., Labarta L., Díaz C., Jiménez A. (2015). Association between serum total antioxidant capacity and mortality in severe septic patients. J. Crit. Care.

[38] Lorente L., Martín M.M., Almeida T., Hernández M., Ferreres J., Solé-Violán J., Labarta L., Díaz C., Jiménez A. (2015). Association between serum substance P levels and mortality in patients with severe sepsis. J. Crit. Care.

[39] Lorente L., Martín M.M., López-Gallardo E., Blanquer J., Solé-Violán J., Labarta L., Díaz C., Jiménez A., Montoya J., Ruiz-Pesini E. (2015). Decrease of oxidative phosphorylation system function in severe septic patients. J. Crit. Care.

[40] Vincent J.L., Moreno R., Takala J., Willatts S., de Mendonça A., Bruining H., Reinhart C.K., Suter P.M., Thijs L.G. (1996). The Sepsis-related Organ Failure Assessment (SOFA) score to describe organ dysfunction/failure. Intensive Care Med..

